# Self-Cleaning Properties of Electrospun PVA/TiO_2_ and PVA/ZnO Nanofibers Composites

**DOI:** 10.3390/nano8090644

**Published:** 2018-08-22

**Authors:** Muhammad Qamar Khan, Davood Kharaghani, Sana Ullah, Muhammad Waqas, Abdul Malik Rehan Abbasi, Yusuke Saito, Chunhong Zhu, Ick Soo Kim

**Affiliations:** 1 Nano Fusion Technology Research Group, Division of Frontier Fibers, Institute for Fiber Engineering IFES- Interdisciplinary Cluster for Cutting Edge Research ICCER, Shinshu University, Tokida 3-15-1, Ueda, Nagano 386-8567, Japan; qamarkhan154@gmail.com (M.Q.K.); kharaghani66@gmail.com (D.K.); Sanamalik269@gmail.com (S.U.); 15f2020e@shinshu-u.ac.jp (Y.S.); 2 Faculty of Textile Engineering, National Textile University, Sheikhupura Road, Faisalabad 37610, Pakistan; waqaskashmiri58@gmail.com; 3 Department of Textile Engineering, Faculty of Engineering, BUITEMS, Quetta 87300, Pakistan; rehan_abbaci@yahoo.com; 4 Faculty of Textile Science and Technology, Shinshu University, Ueda Campus 386-8567, Japan; zhu@shinshu-u.ac.jp

**Keywords:** TiO_2_ & ZnO nanoparticles, PVA, self-cleaning, electrospinning, composite

## Abstract

In this report, polyvinyl alcohol/zinoxide (PVA/ZnO) & polyvinyl alcohol/titanium dioxide (PVA/TiO_2_) nanofibers were manufactured in three different concentrations of ZnO and TiO_2_ NPs for the application of self-cleaning properties because metallic oxides, specifically ZnO & TiO_2_, have the properties to remove the contaminants by hydroxyl radical (OH^−1^), which degrades the contaminants into small molecules and finally into CO_2_ and H_2_O. Therefore, these composites were manufactured by electrospinning. The resultant nanofibers were characterized for morphology by scan electron microscopy (SEM) & transmission electron microscopy (TEM), chemical interactions by Fourier-transform infrared (FT-IR) spectra, crystalline structure by X-ray diffraction (XRD) spectra water absorbency was evaluated by water contact angle, self-cleaning by solar simulator, and thermal degradation was done by thermogravimetric analysis (TGA) for the sake of nanoparticles the content. On the base of the characterization results it was concluded that these PVA/ZnO & PVA/TiO_2_ nanofibers have self cleaning properties, but PVA/ZnO nanofibers have higher self-cleaning properties than PVA/TiO_2_ nanofibers because PVA/ZnO nanofibers have 95% self-cleaning properties, which is higher than PVA/TiO_2_ nanofibers.

## 1. Introduction

In recent times nanofibres have been researched and studied widely because of their unique properties and applications [1]. Particularly, their high surface areas to volume ratio, large length to diameter ratio, and light weightiness have made them suitable for a number of applications [2]. They are widely used for filtration, drug delivery [3], tissue engineering, and sensors application in protective clothing for protection against biological and chemical hazards, food processing, and in microelectronics [4,5]. A number of methods for the production of nanofibers are being used, such as self-assembly, drawing, phase separation, and electrospinning [2]. Among these techniques electorspinning is more versatile due to its simple assembly and ease of production of continuous fibers [1,6].

There are a number of ways through which a self-cleaning property can be imparted to nanofibers [7]. Generally, in macro materials two basic methods that are used to develop self-cleaning surfaces are complete hydophobization or hydrophilization of a surface. In the case of hydrophobic surfaces, water takes away the dust particles and containments that are on the material surface, which has no adhesion because of a reduced wet ability also known as the lotus leaf effect [8,9]. While in case of hydrophilic surfaces developed by coating with semiconducting metal oxides dust, grease and organic containments decompose on exposure to light and are easily taken away by water or rain [10,11,12]. Use of photo-catalytic metallic oxides to develop hydrophilic self-cleaning surfaces is well known. When a photo-catalytic surface is exposed to light of suitable energy it causes the transition of the electrons from the valence band to the conduction band, leaving behind a hole that can react with water to produce hydroxyl radical. Both the hole and hydroxyl radical have very high oxidative power and can destroy organic matters and air pollutants [11].

To develop a nanofiber based photo-catalytic self-cleaning surface nanocomposite is the best option. Polymeric nanocomposites are unique structures that combine the properties of polymers and filler materials and provide improved properties compared to virgin materials [13]. TiO_2_ and ZnO photo-catalysts are being widely used in a number of applications to remove environmental pollutants because of their excellent photo catalytic efficiency and non-toxicity [14,15]. TiO_2_ coated on carbon nanotubes can be used to remove environmental pollutants and to kill microorganisms [16]. ZnO nano particles embedded poly (1,4-cyclohexanedimethylene isosorbide terephthalate) fibers show excellent self-cleaning property [17]. If two exciting photo-catalysts, TiO_2_ and ZnO, are embedded in a suitable polymer solution, then the resultant composite can effectively be used in a number of applications to destroy organic pollutants. In the literature no work is reported regarding the self-cleaning behavior of ZnO and TiO_2_ embedded electrospun PVA nanofibers.

Therefore, in this work we have intended to explore the self-cleaning properties of ZnO and TiO_2_ embedded PVA nanofibers. The use of the economical polymer, PVA, as a base material to develop TiO_2_-PVA and ZnO-PVA nano-composites makes them particularly useful for commercial applications. The resultant nanofibers were characterized by Fourier-Transform Infrared Spectroscope (FT-IR). For the sake of chemical investigation, scanning electron microscope (SEM) was done to investigate the surface morphology, transmission electron microscope (TEM) was done to investigate the dispersion of nanoparticles, and photo-catalytic activity was done to check the self-cleaning properties of the nanofibers.

## 2. Materials and Methods

### 2.1. Materials

ZnO and TiO_2_ nanoparticles (NPs) were purchased from the Sigma-Aldrich Corporation (Louis, MO, USA), with a dispersion concentration of 50.1 wt % and the size of particles was 50 nm. Methylene blue (powder form, soluble 4 mg/4 mL, was purchased from the Sigma-Aldrich Corporation (Louis, MO, USA). Poly vinyl alcohol (MW: 85,000–124,000, 87–89% hydrolyzed) was purchased from the Sigma-Aldrich Corporation (Louis, MO, USA). Glutaraldehyde (GA, 50% in aqueous solution) was purchased from MP Biomedical (Tokyo, Japan).

### 2.2. Method

**Figure 1:**
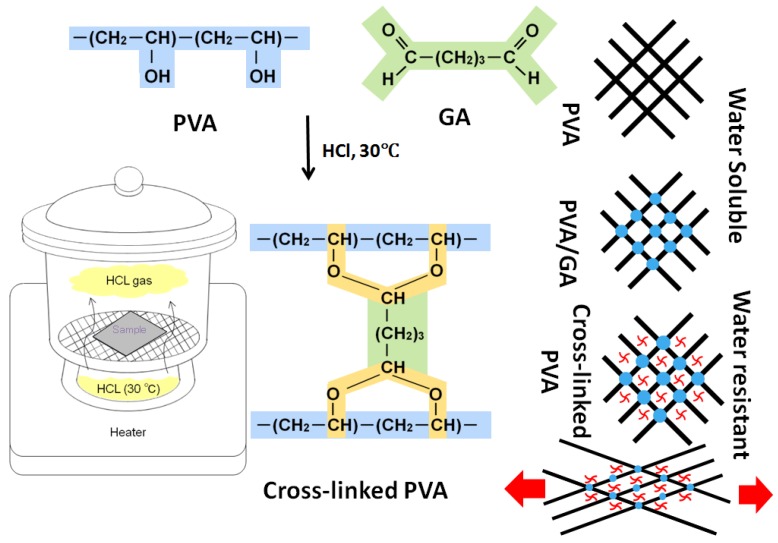
Illustration scheme of cross-linking nanofibers.

PVA 10% by weight was dissolved in de-ionized H_2_O at 60 °C and stirred for 12 h. Glutaraldehyde was added in the solution at 2.5 wt % for cross-linking of the solution. Nanoaprticle suspensions of ZnO & TiO_2_ were separately embedded in that PVA solution to fabricate the PVA/ZnO and PVA/TiO_2_ nanofibers. For the fabrication of PVA/ZnO nanofibers three different concentrations; 5 wt %, 7 wt % and 9 wt % of ZnO nanoparticles were blended with PVA solution by stirring for 5 h and similarly to fabricate the PVA/TiO_2_ nanofibers, TiO_2_ nanoparticles in three different concentrations, 5 wt %, 7 wt %, and 9 wt %, were blended with PVA solution by stirring for 5 h. The electrospinning of PVA/ZnO and PVA/TiO_2_ solutions was done as the solution was loaded in the plastic syringe with an inner diameter of 0.60 mm, in which a Cu electrode was adjusted. The distance from capillary tip to collector was 15 cm and the supply of voltage was 12 kV. The PVA, PVA/ZnO, and PVA/TiO_2_ nanofibers were formed without beads at room temperature and at 45% humidity. The resultant nanofibers were cross linked to form nanofibers that are water resistant yet can absorb water but are not soluble in water. The illustration scheme of cross-linking is shown in Figure 1. The hydrochloric acid (HCL) foaming of PVA/ZnO/GA & PVA/TiO_2_/GA was done at 30 °C for 60 s. In this reaction, GA and HCl acted as a chemical cross-linking agent and a catalyst, respectively. To enhance the crystalline structure and for stable cross-linking, the PVA/ZnO nanofibers were kept at −20 °C for 3 h, which was followed by thawing at 15 °C for 3 h. This process was repeated three times and the resultant nanofibers were dried at room temperature for 24 h, and then estimated.

### 2.3. Characterizations

The morphology of the PVA, PVA/ZnO, and PVA/TiO_2_ nanofibers was investigated by scan electron microscopy (SEM) (JSM-5300, JEOL Ltd., Tokyo, Japan) accelerated with voltage of 12 kV and transmission electron microscopy (TEM) (JSM-5300, JEOL Ltd., Tokyo, Japan) accelerated with 200 kV. The chemical interactions were studied by FT-IR (IR Prestige-21 by shinmadzu, Nagano, Japan), Wide angle X-ray diffractions (WAXD) spectra were performed for the evaluation of the crystal structure at 25 °C with nanofiber samples using a Rotaflex RT300 mA (Rigaku manufacturer, Osaka, Japan) and Nickel-filtered Cu. Ka radiation was used for measurements, along with an angular angle of 5 ≤ 2θ ≤ 50°. Photo-catalytic activity was done by a solar simulator (XES-40S3, San-ei Electric, Nagano, Japan), stress-strain behavior was studied by a tensile strength tester (Universal Testing Machine, Tenilon RTC 250 A, A &D company Ltd., Tokyo, Japan), water contact angle measurements were done by a contact angle meter (Digidrop, GBX, Whitestone way, France) and photo-catalytic activity was done by a solar simulator (XES-40S3, San-ei Electric, Nagano, Japan). The light intensity was 1000 W/m^2^, the wavelength range was 350–1100 nm, and the self-cleaning efficiency was calculated as the following Equation (1).

(1) $$\mathrm{Degradation}\;(\%)\;=\frac{\mathrm{Ii}-\mathrm{Id}}{\mathrm{Ii}}\times 100$$

Thermal degradation of PVA, PVA/ZnO & PVA/TiO_2_ was performed by thermogravimetric analysis. This thermogravimetric analysis was performed on the thermo-plus TG 8120 (Rigaku Corporation, Osaka, Japan). It was operating in a static mode under air atmosphere at a heating rate of 10 °C/min and a temperature range of 0–700 °C.

## 3. Results and Discussion

### 3.1. Morphology of Nanofibers

**Figure 2:**
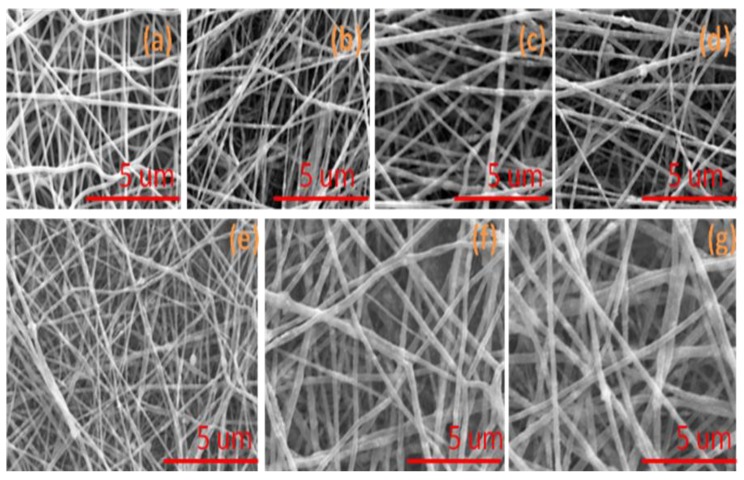
Scanning electron microscope (SEM) images of (**a**) Neat PVA nanofibers; (**b**) 5% PVA/ZnO nanofibers; (**c**) 7% PVA/ZnO nanofibers; (**d**) 9% PVA/ZnO nanofibers; (**e**) 5% PVA/TiO_2_ nanofibers; (**f**) 7% PVA/TiO_2_ nanofibers; (**g**) 9% PVA/TiO_2_ nanofibers.

In order to investigate the surface morphology of nanofibers, SEM images were analyzed as mentioned in Figure 2. All PVA, PVA/ZnO, and PVA/TiO_2_ nanofibers were bead free and PVA/ZnO and PVA/TiO_2_ nanofibers showed that the blending of the nanoparticles did not affect the surface morphology of the PVA nanofibers as mentioned in Figure 2b–g. It was also analyzed that both ZnO and TiO_2_ nanoparticles did not change the surface morphology of the PVA nanofibers. Both nanoparticles affected the size of the nanofibers. When the concentration of nanoparticles was increased, the size of the nanofibers was also increased, as shown in Figure 2b–g.

**Figure 3:**
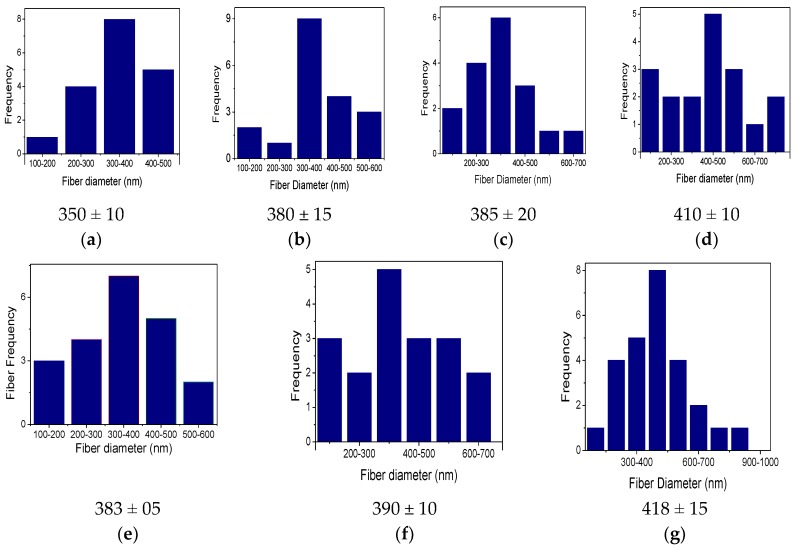
Average diameter distributions analysis of (**a**) Neat PVA nanofibers; (**b**) 5% PVA/ZnO nanofibers; (**c**) 7% PVA/ZnO nanofibers; (**d**) 9% PVA/ZnO nanofibers; (**e**) 5% PVA/TiO_2_ nanofibers; (**f**) 7% PVA/TiO_2_ nanofibers; (**g**) 9% PVA/TiO_2_ nanofibers.

In order to investigate the effect of ZnO & TiO_2_ nanoparticles on the average diameter of the PVA nanofibers, distribution graphs were studied, as shown in Figure 3. It was analyzed that the average diameter of PVA/ZnO and PVA/TiO_2_ was affected by the blending of ZnO & TiO_2_ nanoparticles. The average diameter of neat PVA nanofibers was 350 ± 10 nm but the average diameter of PVA/ZnO nanofibers was increased as the concentration of ZnO NPs was increased as mentioned in Figure 3b–d; 5%, 7% & 9% PVA/ZnO nanofibers have an average diameter of 380 ± 15, 385 ± 20, and 410 ± 10 nm, respectively. The average diameter of PVA/TiO_2_ nanofibers was also increased when the concentration of TiO_2_ was increased, as mentioned in Figure 3e–g; 5%, 7% & 9% PVA/TiO_2_ nanofibers have an average diameter of 383 ± 05, 390 ± 10 and 418 ± 15 nm, respectively. It showed that PVA/TiO_2_ nanofibers have a greater average diameter than the PVA & PVA/ZnO nanofibers. It also means that the TiO_2_ NPs have a greater effect on the PVA nanofibers than ZnO NPs.

**Figure 4:**
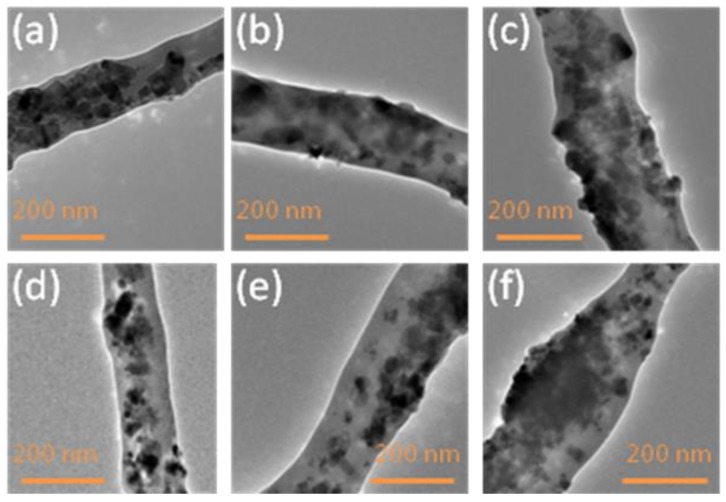
Transmission electron microscope (TEM) analysis of (**a**) 5% PVA/ZnO nanofibers; (**b**) 7% PVA/ZnO nanofibers; (**c**) 9% PVA/ZnO nanofibers; (**d**) 5% PVA/TiO_2_ nanofibers; (**e**) 7% PVA/TiO_2_ nanofibers; (**f**) 9% PVA/TiO_2_ nanofibers.

In order to investigate the dispersion of the ZnO and TiO_2_ nanoparticles on the PVA nanofibers, TEM images were analyzed, as shown in Figure 4. It was analyzed that there was uniform and well dispersion of both the ZnO and TiO_2_ nanoparticles on the PVA nanofibers. TEM images also showed that when the concentrations of nanoparticles were increased, then the diameters of PVA/ZnO and PVA/TiO_2_ were also increased. When the concentration of NPs was increased the dispersion of NPs on PVA nanofibers also was increased, as mentioned in Figure 4.

### 3.2. Chemical Structure Analysis

**Figure 5:**
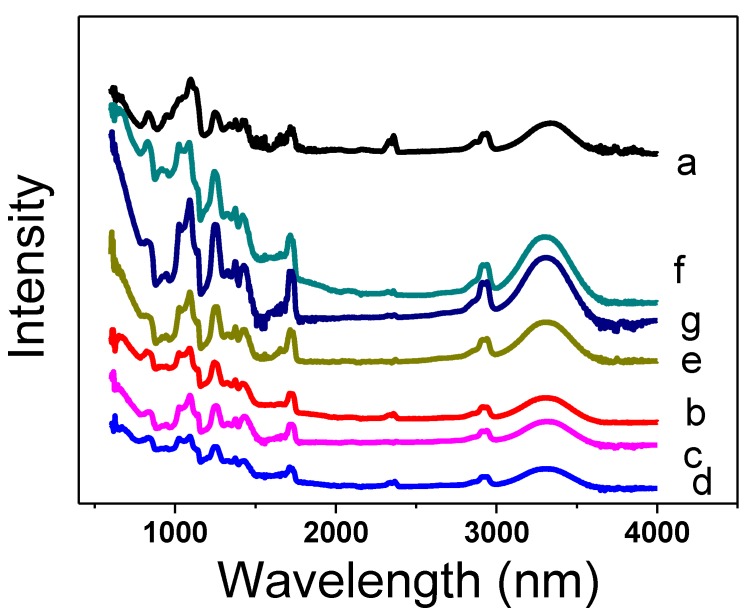
Fourier-transform infrared (FTIR) spectra of (**a**) Neat PVA nanofibers; (**b**) 5% PVA/ZnO nanofibers; (**c**) 7% PVA/ZnO nanofibers; (**d**) 9% PVA/ZnO nanofibers; (**e**) 5% PVA/TiO_2_ nanofibers; (**f**) 7% PVA/TiO_2_ nanofibers; (**g**) 9% PVA/TiO_2_ nanofibers.

In order to investigate the chemical structure analysis of PVA, PVA/ZnO, and PVA/TiO_2_ nanofibers, ATR-FTIR spectra were studied, as shown in Figure 5. It was analyzed that there were successful chemical interactions of functional groups between PVA and ZnO & TiO_2_ NPs, as shown in Figure 5b–g. The spectra showed that in the neat PVA nanofibers, the bands at about 3320, 2940, 1437, 1093, and 850 cm^−1^ showed the vibrations of –OH, –CH_2_CH, C–C & C–O functional groups of PVA, respectively [18]. The new intense broad bands at 1100 cm^−1^ to 1000 cm^−1^ and 750 cm^−1^ are assigned to metal oxide of the of Zn & Ti, because these oxides have a moderate dipole moment. The oxides of Zn & Ti with more than one oxygen atom bound to a single metal atom usually absorb in the region 1100 to 750 cm^−1^. In this spectra of PVA/ZnO and PVA/TiO_2_, it was shown that O–H bending at 1579 cm^−1^ and O–H stretching at 3320 cm^−1^ occurred, which was due to the –O bonding of TiO_2_ & ZnO with the –H of PVA. There was stretching vibration of –O of TiO_2_ & ZnO with –C and –O of PVA at 1050 and 850 cm^−1^, respectively, as shown in Figure 5 [19,20,21]. Therefore, these spectra confirmed that this self cleaning composite was composed with PVA/ZnO and PVA/TiO_2_.

### 3.3. XRD Study

**Figure 6:**
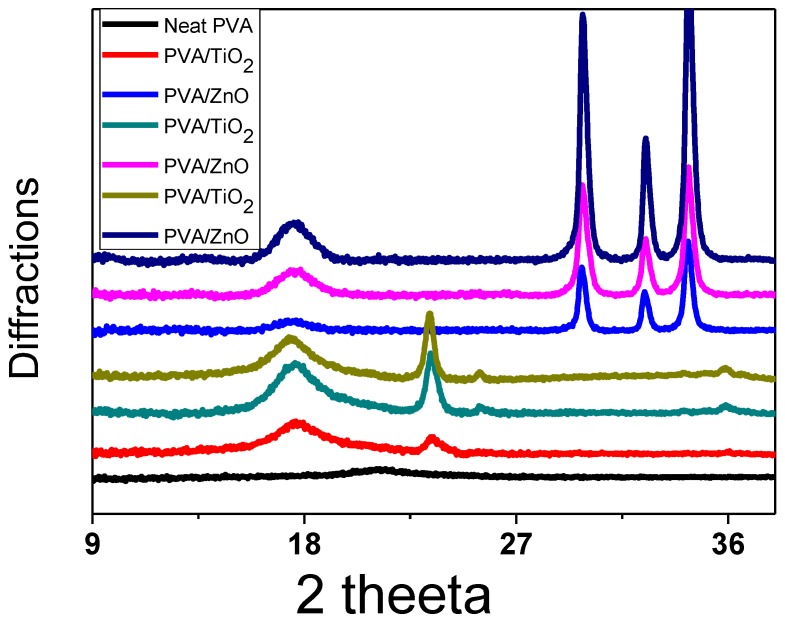
X-ray diffraction (XRD) spectra of Neat PVA nanofibers and PVA/ZnO nanofibers.

In order to investigate the crystalline structure of PVA, PVA/ZnO and PVA/TiO_2_ nanofibers XRD spectra were studied, as shown in Figure 6. It was analyzed that the crystallinity of the PVA nanofibers was affected by blending the nanoparticles of ZnO and TiO_2_, but ZnO NPs had a greater effect than TiO_2_ NPs, as shown in Figure 6. PVA/ZnO nanofibers have a greater crystalline structure than PVA & PVA/TiO_2_ nanofibers. 9% by weight ZnO/PVA nanofibers have a higher crystalline structure than other 7% & 5% by weight ZnO/PVA nanofibers, which exhibit the peak at 18°, 29°, 31° & 34° with high intensity, 7% & 5% by weight ZnO/PVA nanofibers exhibit the peak at at 18°, 29°, 31° & 34° with low intensity than 9% by weight ZnO/PVA nanofibers. 9% by weight TiO_2_/PVA nanofibers exhibit the peak at 18°, 20° and 36° with high intensity than 5% & 7% by weight of TiO_2_/PVA nanofibers but 5% by weight of TiO_2_/PVA nanofibers did not show the crystalline structure at 36° [15,19]. Therefore, due to cross-linking, the crystalline structure of PVA/ZnO & PVA/TiO_2_ was increased. This crystalline structure made them water resistant nanofibers.

### 3.4. Water Contact Angle Measurements

**Figure 7:**
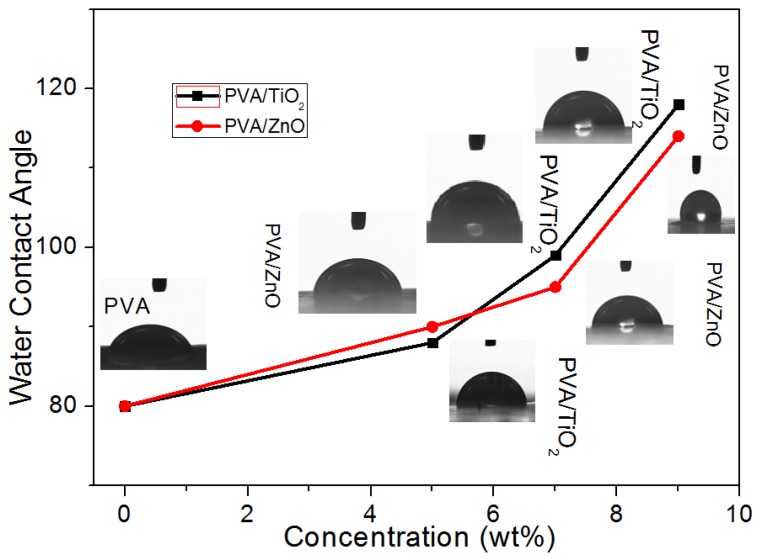
Wetting behavior of Neat PVA nanofibers; 5% PVA/ZnO nanofibers; 7% PVA/ZnO nanofibers; 9% PVA/ZnO nanofibers; 5% PVA/TiO_2_ nanofibers; 7% PVA/TiO_2_ nanofibers; 9% PVA/TiO_2_ nanofibers.

In order to investigate the wetting behavior of PVA, PVA/ZnO and PVA/TiO_2_ nanofibers, water contact angle measurements were studied, as shown in Figure 7. It was analyzed that when crystallinity of PVA/ZnO & PVA/TiO_2_ nanofibers was increased, the capacity of water absorbency was reduced. Therefore, these nanofibers showed more water contact angle values than neat PVA nanofibers, as mentioned in Figure 7. It was also analyzed that when the concentration of both ZnO and TiO_2_ NPs was increased the water contact angle’s value was increased, but TiO_2_ NPs has a greater effect than ZnO NPs, as mentioned in Figure 7.

#### 3.4.1. Photo-Catalysis Study

**Figure 8:**
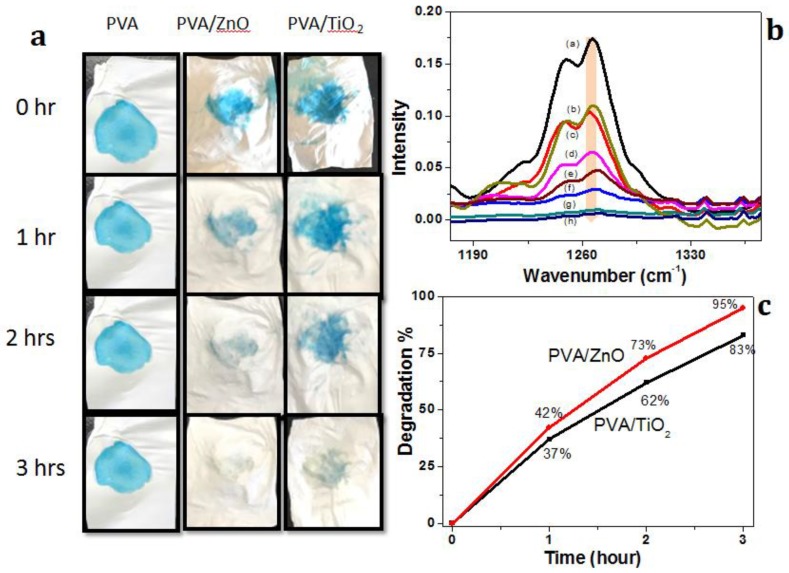
Photo-catalysis efficiency in 3 h of neat PVA nanofibers, 9% PVA/ZnO nanofibers and 9% PVA/TiO_2_ nanofibers, (**a**) photocatalytic activity by solar simulator; (**b**) Self-cleaning efficiency calculated by FT-IR; (**c**) profile of self cleaning efficiency of PVA/ZnO & PVA/TiO_2_.

In order to investigate the self-cleaning efficiency of PVA, PVA/ZnO and PVA/TiO_2_ nanofibers, a photo-catalysis study was conducted by solar simulator with a light intensity of 1000 W/m^2^ and a wavelength range of 350–1100 nm. In this activity, the photo-catalyst absorbs the UV light, which convert the H_2_O into hydroxyl radical (OH^−1^), which degrades the contaminants into small molecules and finally into CO_2_ and H_2_O [17]. The objective of this research was to develop the self-cleaning nanocomposites in two different ways: PVA/ZnO and PVA/TiO_2_ nanofibers. Three different concentrations, 5%, 7% & 9% by weight, of ZnO and TiO_2_ NPs were separately blended in PVA nanofibers, but the self-cleaning comparison of both was analyzed at 9% by weight PVA/ZnO and PVA/TiO_2_ nanofibers, as shown in Figure 8. Methylene blue (MB) dye was used for analyzing the self-cleaning properties of these composites. 2 μL MB was dropped on each sample of PVA, PVA/ZnO & PVA/TiO_2_ nanofibers. It was observed that there was no self-cleaning property in PVA but PVA/ZnO and PVA/TiO_2_ nanofibers have self-cleaning properties, as mentioned in Figure 8. The comparative study between PVA/ZnO and PVA/TiO_2_ nanofibers was done in two ways; first by the naked eye, as shown in Figure 8A, and second by ATR spectra, in which the intensity of the methylene blue was measured by using Equation (2).

(2) $$\mathrm{Degradation}\;(\%)\;=\frac{\mathrm{Ii}-\mathrm{Id}}{\mathrm{Ii}}\times 100$$

where Ii is the initial intensity of dyed nanofibers and Id is the degraded intensity of dyed nanofibers. Therefore, degradation efficiency/percentage was calculated from ATR spectra, which showed that PVA/ZnO nanofibers have a higher self-cleaning efficiency than PVA/TiO_2_ nanofibers because PVA/ZnO nanofibers showed a 95% self-cleaning efficiency within three hours but the PVA/TiO_2_ nanofibers showed less efficiency than the PVA/ZnO nanofibers, as shown in Figure 8b,c.

#### 3.4.2. Thermogravimetric Analysis (TGA)

**Figure 9:**
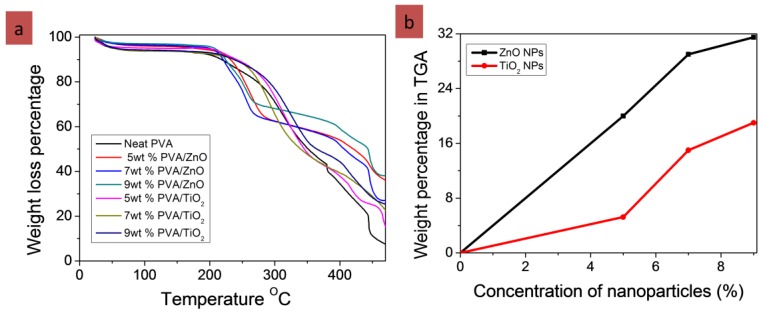
Results of TGA (**a**) showed the degradation behavior; (**b**) content percentage of ZnO and TiO_2_ NPs in 6 mg of sample.

Thermal degradation of PVA, PVA/ZnO & PVA/TiO_2_ was performed by thermogravimetric analysis as shown in Figure 9. This thermogravimetric analysis was performed on the thermo-plus TG 8120 Rigaku Corporation Japan. It was operating in a static mode under air atmosphere at a heating rate of 10 °C/min and a temperature range from 30–500 °C. The samples weighing 6 mg were placed in the pans. The TGA curves of neat PVA, PVA/TiO_2_ and PVA/ZnO nanofibers as shown in Figure 9 showed that PVA/TiO_2_ & PVA/ZnO showed the same thermal stability as neat PVA nanofibers. In this test PVA nanofibers did show smaller residual mass than PVA/TiO_2_ & PVA/ZnO nanofibers. The PVA/TiO_2_ & PVA/ZnO nanofibers showed the residual mass that confirmed the presence of nanoparticles of ZnO and TiO_2_ as shown in the Figure 9. The neat PVA showed 93% degradation at 470 °C, but 5 wt % PVA/ZnO showed 73%, 7 wt % PVA/ZnO 64%, and 9 wt % PVA/ZnO 61% showed degradation, which confirmed that there was a good amount of ZnO nanoparticles. Similarly, PVA/TiO_2_ also showed less degradation than PVA, which confirmed the presence of TiO_2_ nanoparticles in the PVA/TiO_2_ as shown in Figure 9B [22,23].

## 4. Conclusions

Herein, we successfully developed self-cleaning PVA/ZnO & PVA/TiO_2_ nanofibers by electrospinning in three different concentrations of ZnO and TiO_2_ NPs. On the basis of the characterization results, it was concluded that these PVA/ZnO & PVA/TiO_2_ nanofibers have self-cleaning properties, but PVA/ZnO nanofibers have higher self-cleaning properties than PVA/TiO_2_ nanofibers because 9% by weight PVA/ZnO nanofibers has 95% self-cleaning properties, which is higher than 9% by weight PVA/TiO_2_ nanofibers. This innovative research is essential to form intelligent textiles for stain eliminating from non-washable & non-woven products, such as surgical gown, wound dressings, and home textiles. This research will be fruitful to fulfill the stated and implied needs of customers.
